# Cone-rod dystrophy and a frameshift mutation in the *PROM1* gene

**Published:** 2009-08-28

**Authors:** Eran Pras, Almogit Abu, Ygal Rotenstreich, Isaac Avni, Orit Reish, Yair Morad, Haike Reznik-Wolf, Elon Pras

**Affiliations:** 1 Department of Ophthalmology, Assaf Harofeh Medical Center, Zerifin, Israel; 2 Gartner Institute of Human Genetics, Sheba Medical Center, Tel Hashomer, Israel; 3 Goldschleger Eye Research Institute, Sheba Medical Center, Tel Hashomer, Israel; 4 Institute of Human Genetics, Assaf Harofeh Medical Center, Zerifin, Israel; 5 Sackler Faculty of Medicine, Tel Aviv University, Israel

## Abstract

**Purpose:**

To identify the genetic cause underlying autosomal recessive cone-rod dystrophy (CORD) and high myopia.

**Methods:**

Nine members of a consanguineous Arab family were clinically examined and were given fluorescein angiography (FA), biometry, and full field electroretinogram (ERG) testing. Blood samples were collected for DNA extraction. A homozygousity genome-wide scan was performed using >382 polymorphic microsatellite markers on genomic DNA from three affected family members. Regions of homozygosity were further analyzed in all members of the family. Mutation analysis of the *PROM1* gene was performed by direct sequencing of PCR-amplified exons.

**Results:**

The phenotype is characterized by severe visual impairment evident in the first decade of life. Affected family members have bull`s-eye macular appearance, peripheral retinal pigment clumps, and cone-rod type ERG changes. Additionally, they have high myopia with axial lengths exceeding 25.3 mm. A genome-wide scan detected a region of 2.1 Mb on chromosome 4p that fully segregates with the disease within the family. This region encompasses the *PROML1* gene, mutations of which have been implicated in retinal dystrophies. *PROML1* mutation analysis identified a novel single nucleotide insertion at position 1629 of the cDNA resulting in truncation of approximately one-third of the protein.

**Conclusions:**

The mutation described in this report further expands the clinical spectrum of *PROM1* mutations.

## Introduction

Retinal dystrophies display a high degree of clinical and genetic heterogeneity. Frequently, a single disease may be caused by mutations in different genes, and in some cases, mutations in a single gene may lead to clinically distinct diseases. An example of the latter are mutations in the *ABCA4* gene, which were previously associated with Stargardt disease (STGD; OMIM 248200), retinitis pigmentosa (RP; OMIM 268000), and cone-rod dystrophy (CORD; OMIM 604116 or 120970) [1]. CORDs are progressive retinal disorders, characterized by simultaneous involvement of both cone and rod photoreceptor cells [2-4]. They are heterogeneous in terms of clinical manifestations, the hereditary pattern, and causative genes (RETNET) [5,6]. Significant differences in onset age and disease severity have been documented even within a single family [7]. Usually they present at childhood with cone dysfunction-related symptoms, including decreased visual acuity, photophobia, impaired color vision, and nystagmus. With time, poor night vision and restricted peripheral visual fields develops, reflecting rod photoreceptor involvement. Typical fundus changes in CORDs in the early stages are characterized by macular atrophy and retinal pigmenent epithelium changes. However with disease progress, pigmentation resembling RP appear in retinal periphery. Full field electroretinography (ERG) testing is abnormal with either cone (photopic) responses more reduced than rod (scotopic) responses, or equally reduced cone and rod systems [2-7].

PROM1 (OMIM 604365) is a membrane glycoprotein specifically concentrated in various membrane structures that protrude from the planar areas of the plasmatic membrane. It binds to the plasma membrane cholesterol and is associated with a particular membrane microdomain in a cholesterol-dependant manner [8]. It has a unique membrane topology with five transmembrane spanning segments and two large N-glycosylated extracellular loops [9]. Several splice variants affecting the protein sequence have been identified with a broad range of expression [10]. Yet it is on the visual system where interference with its function has the most obvious effect. PROM1 is expressed in both types of photoreceptors. Mutations have previously been implicated in both RP families where rod dysfunction predominates [11,12] and in macular dystrophy families where cones are mostly affected [13]. Mounting evidence assigns a critical role for PROM1 in the morphogenesis of new disc membranes in photoreceptor’s outer segments [11-13]. We describe a consanguineous Arab family who has a novel PROM1 mutation that functionally disrupts both types of photoreceptors and presents as cone-rod dystrophy.

## Methods

### Patients and methods

**Figure 1:**
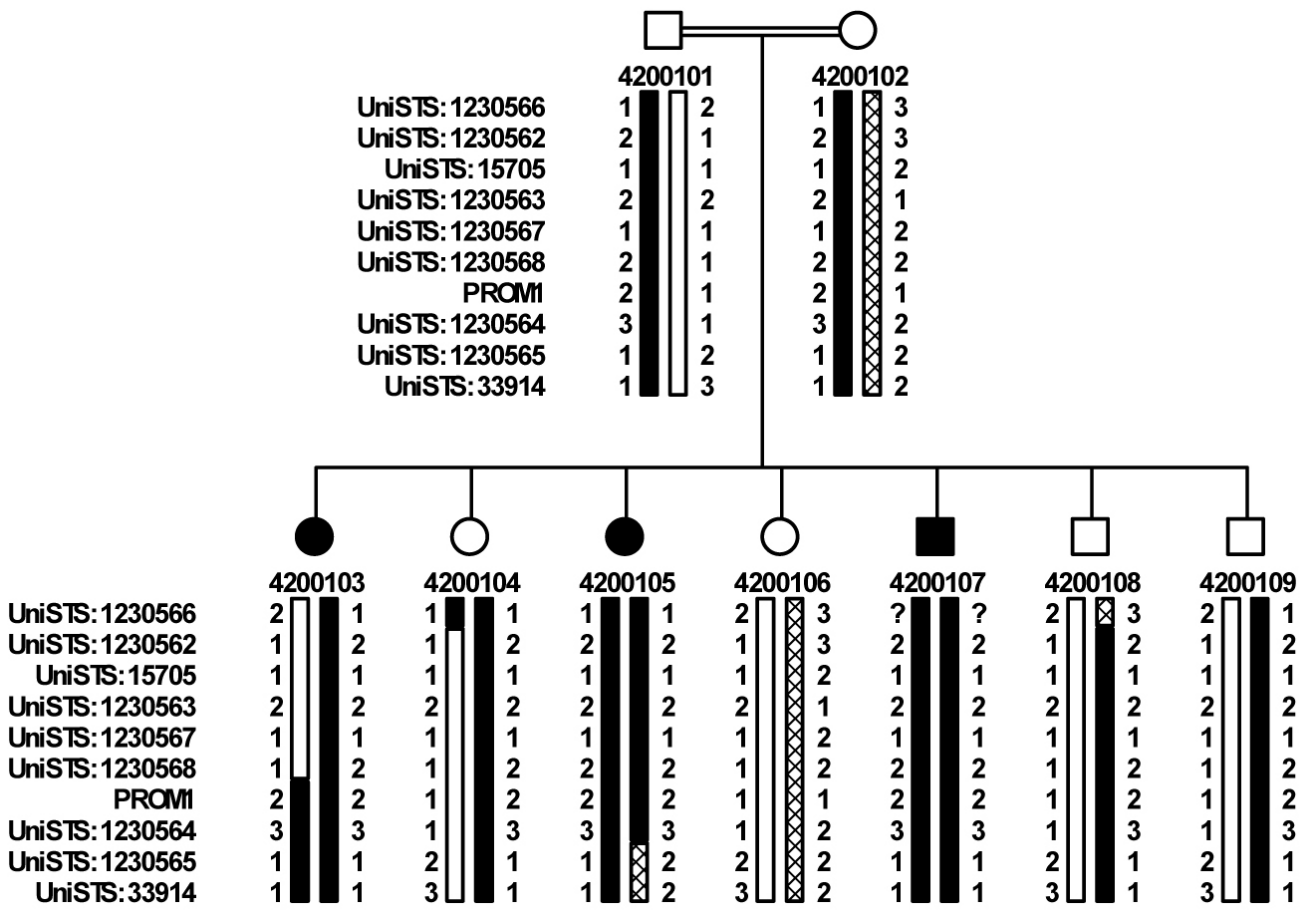
Family pedigree and haplotypes surrounding the PROM1 locus. Shown are nine chromosome 4 microsatellite markers allele readings, and the *PROM1* c.1349insT change (allele 2). The marker order is to the left of each generation. Filled symbols indicate affected individuals; open symbols indicate unaffected individuals; and filled bars indicate carrier haplotypes.

A consanguineous Arab family (Pedigree 42001) was ascertained at Assaf Harofeh Medical Center (Figure 1). The parents are first degree cousins and three of their seven children suffer from severe visual impairment evident at the first year of life. Subsequent ophthalmic examinations led to the diagnosis of CORD. The protocol of the study adhered to the provisions of the Declaration of Helsinki, and after obtaining informed consent from the participants, DNA was extracted from peripheral blood using a commercial kit (Gentra System Inc., Minneapolis, MN). Fifty DNA samples of Israeli Arab subjects without any known ocular diseases were used as controls.

### Clinical assessment

A full medical history was obtained and standard ophthalmologic examinations including best-corrected visual acuity measurements, slit-lamp biomicroscopy and color vision tests were performed in all family members. The patients also underwent kinetic visual fields, color fundus photography, flourescein angiography (FA), and full-field ERG scans which were obtained from both eyes of each patient by following the protocol of the International Society for Clinical Electrophysiology of Vision (ISCEV; LKC Technologies, Gaithersburg, MD) [14]. The diagnosis of CORD in this study was based on the following criteria: reduced visual acuity and nystagmus evident at infancy, impairment of color vision, fundoscopic evidence of maculopathy with peripheral retinopathy, and the demonstration of a cone-rod pattern ERG.

### Molecular biology

A homozygosity genome-wide scan was performed on DNA samples from the three affected individuals using a commercial genotyping service (Laboratory of DNA Analysis at the Institute of Life Sciences, Hebrew University of Jerusalem). A total of 382 microsatellites spaced at approximately 10 cM intervals were analyzed using the MD-10 linkage mapping set (ABI MD-10; Applied Biosystems, Foster City CA). Regions that showed homozygous readings in the initial scan were further evaluated with additional polymorphic markers, for segregation in the entire family as previously described [15]. The entire coding region and exon-intron boundaries of the *PROM1* gene were amplified using 25 pairs of primers (Table 1), and sequenced with ABI BigDye Terminator cycle sequencing kit v3.1 (Applied Biosystems) according to manufacturer’s instructions. The mutation was confirmed and analyzed on an automated ABI Prism 3100 Genetic Analyzer (Perkin Elmer, Waltham, MA) using the fluorescent primer pairs 5′-CTA ACA CTG TGC TTG CCT CTC-3′ and 5′-ACT CAC ACC ATG AGG AAG ACG-3′.

**Table 1:** Forward and reverse primers for PCR amplification of *PROM1* exons.

**Exon number**	**Forward primer (5′-3′)**	**Reverse primer (5′-3′)**
1	GAAGATTCAGCAGATCCAGTGCT	CATTCCTCGCAACCTATGTAACC
2	TGGCTTCTGGCTAGAGGTCATTA	TGGTTCAAATGGGATTTGTAAGG
3	TTCCTCCTTGTGGGATATGAATG	GGAACAAGATACGGCATTTCAAG
4	AAAAGAACTTTGTACACCATGGAATG	GGCAGCTTCATTACAACGCTAAT
5	TGAGTCCTGTTTTGTAGCCCCTA	CACCAGTCTACGCTGATTCACTG
6	GCTGGTTACCTGAAAATGTCCTG	GACACATTGGCAATAAGGCTAGG
7	TCAAGATGATAACACCATGCTCCT	ATCATCTGAAATGGCAACAGCTT
8	TGGAAGAGTGGAGCTAGTTGGAG	CTTTTACTCCTTTGCTCCTGCTG
9	CAAAAGAAACTGCGATTGTACCC	CTTAGCATGCCACTTCACACATC
10	GGGACCCCCTATATGAAAAACTTC	TCCGAATGACACAATTGTAAAGC
11	TAAAGTCAGTGCTCACAGCTTGC	ATGCGAACCTTCTATGCATTGTT
12	ACCAGGAACAATGCAAACCTAGA	GGCTTGACAGAAGTACCCAAATC
13	AGGTGGATGATCTGTTTCACCTG	AACTGCTTATAAGTTTGCACTGCTCT
14	GTTGGAAATCAACCAGAAAAATAATG	CCAGAGATTATTGGAGAGCGAGA
15	CAAGGCAAGAAGTCAGAAGTGGT	CGTTTTGGAACCAAATAGAGGTG
16	CCACATCCAGCTTTTATTGCTCT	GAAGTAGTTGTGCAGCACTGTGAA
17	CAAATGTTGCCACCTGTTTAAGA	GACGGAAACGTAATGACACACAC
18	GAGAGTCCATGGTTCTGTGCTTT	CAGAGGGAGGTGCAATTATTTTG
19	TTTGATGGCTATCTTGTGGGAAG	CCTGCTAAGATGAGGTCTGCACT
20–21	GGTGTTGCAGAGCTGAGTTACAG	AAGTCTTGGTCCTGCACATCAAT
22	TATCCATCTGTGACCCAGGAGTT	CGCCCAGAACTTTGACTTTTCTA
23	CTGGTCCACATGACATTCTCAAA	ACAGCACCACCTAGAAAATGACC
24–25	TTTGCACTGTCAGTATCCGTGTT	ACTCATGGCATCATGGAACACTA
26	ACCTTTAGTCACATGCCTGCTTC	AGGTACAGAGGGTGGACTGGAC

## Results

### Clinical examination

**Figure 2:**
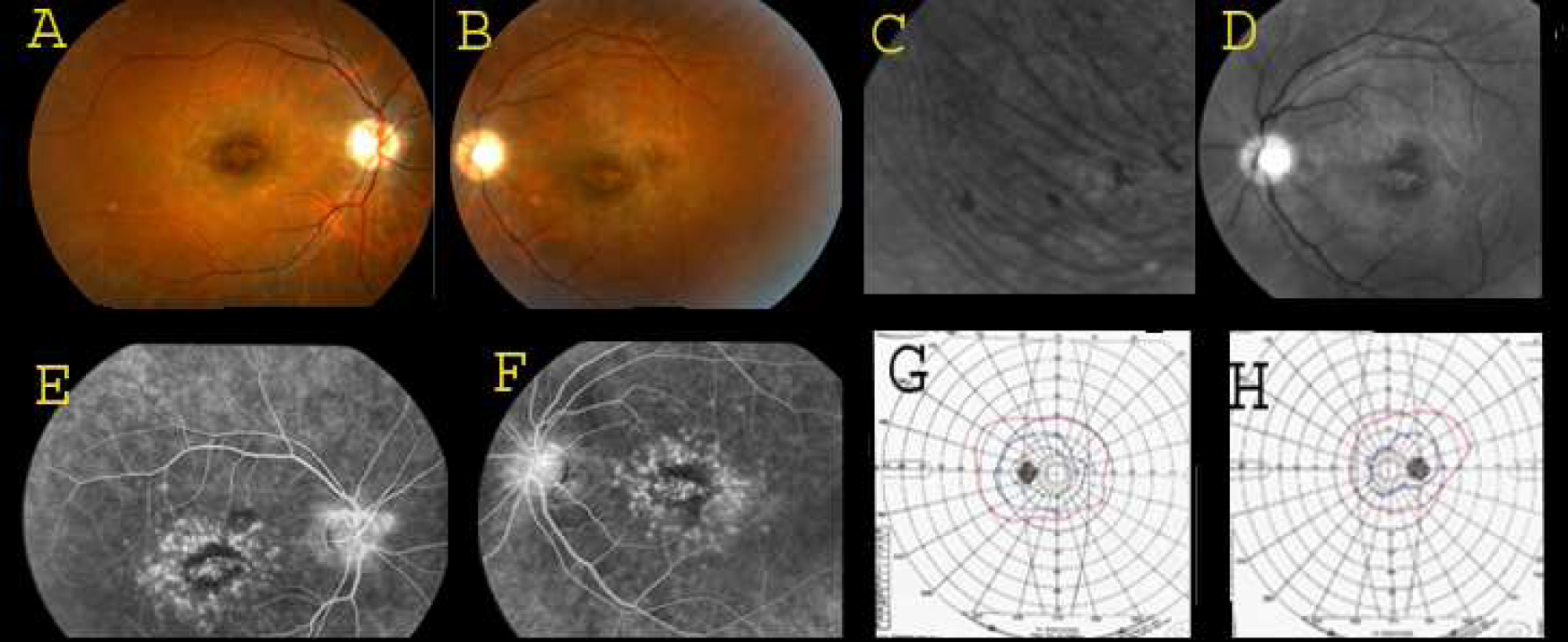
Clinical finding’s in CORD family 42001. Clinical pictures from affected family members showing classical features of CORD. Fundus photographs from individual 4200103 aged 19. Bull’s-eye macular atrophy is shown in photographs **A** (right eye), **B** (left eye), and **D** (left eye “red-free” photograph). The macula in this individual has a peri-foveal ring of RPE hypertrophy bordered centrally and peripherally with RPE atrophy. Fluorescein angiogram imaging demonstrating a ring of block-fluorescence by the RPE hypertrophy, bordered centrally and peripherally by hyper-fluorescence, due to RPE atrophy (“window-defect”; right eye- **E**, and left eye- **F**). “Red-free” imaging of peripheral retina, demonstrates pigment clumps (**C**). Goldman perimetry from family member 4200105 exhibiting concentric constriction of visual fields to 40 degrees temporally, and 30 degrees nasally. Red isopter’s stimulus is IVe4, blue isopter’s stimulus is IVe3 and a green isopters stimulus is IVe2 (Left eye **G** and right eye **H**).

**Figure 3:**
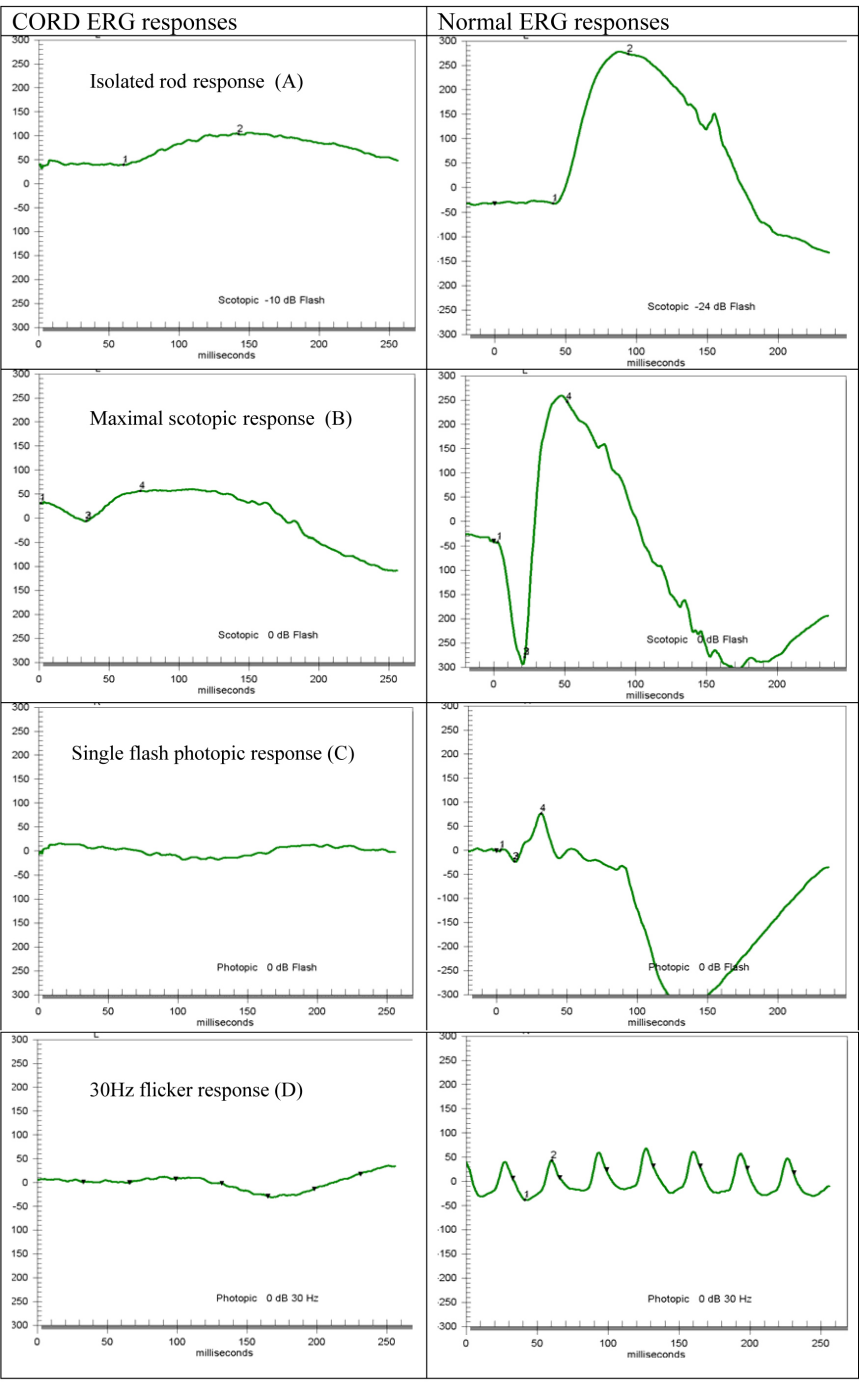
Electroretinogram studies. Shown are scotopic and photopic ERG responses from one affected family member (4200105; left column) and a normal control (right column). The cone-derived responses in the affected family member revealed neither detectable single flash photopic response nor flicker 30 Hz response(**C**, **D**). Assessment of the rod-derived functions by the scotopic single flash test show a detectable but markedly abnormal respond, (**A**, **B**). These findings are consistent with CORD.

The ocular examination in all three affected patients revealed early macular involvement accompanied by growing rod-related dysfunction and high myopia (Table 2). Central visual dysfunction was apparent at early childhood manifested by nystagmus, mild photophobia, color vision deficiency, and poor visual acuity ranging from finger counting to 6/60. None of these findings were found in the parents or unaffected siblings. Night vision deteriorated in the late teens. The degree of the myopia was high with axial lengths exceeding 25.3 mm. On fundus examination and FA, changes consistent with bull’s-eye macular degeneration (Figure 2A,B,D,E) were documented as early as 15 years of age, whereas retinal periphery appeared to be preserved with only occasional pigment deposits (Figure 2C). Goldman visual field testing demonstrated constricted peripheral visual field isopters and reduced central sensitivity (Figure 2F-G). These are in line with the full field ERG findings, which showed nondetectable photopic single flash or flicker 30 Hz stimulus responses representing severe cone-derived functions. Rod-derived measurements under scotopic backgrounds revealed a residual but markedly abnormal result (Figure 3). There was no evidence of keratoconus, hearing loss, polydactyly, or any systemic abnormalities. Taken together, the association of symptoms, ophthalmoscopy findings, and visual function tests were all consistent with CORD and high myopia.

**Table 2:** Clinical details on affected individuals in family 42001

**ID**	**Gender**	**Age** **(years)**	**Onset age** **(years)**	**Visual acuity (OD;OS)**	**Myopia** **(Axial length; OD;OS)**	**Nystagmus**
4200103	F	19	Early childhood	CF; CF	−11 dpt (26 mm); −10 dpt (25.8 mm)	Yes
4200105	F	25	Early childhood	CF; CF	−8.0 dpt (25.3 mm); −9.0 dpt (25.5 mm)	Yes
4200107	M	29	Early childhood	6/120; 6/60	−10 dpt (25.5 mm); −10 dpt (25.5 mm)	Yes

The table provides information regarding the symptoms experienced by the affected CORD patients. Three family 42001 individuals suffer from severe impairment of visual acuity, nystagmus, and high axial myopia evident at early childhood. Abbreviations: diopters (dpt); counting fingers at a distance ranging from 1 to3 m (CF).

### Segregation analysis and mutation detection

**Figure 4:**
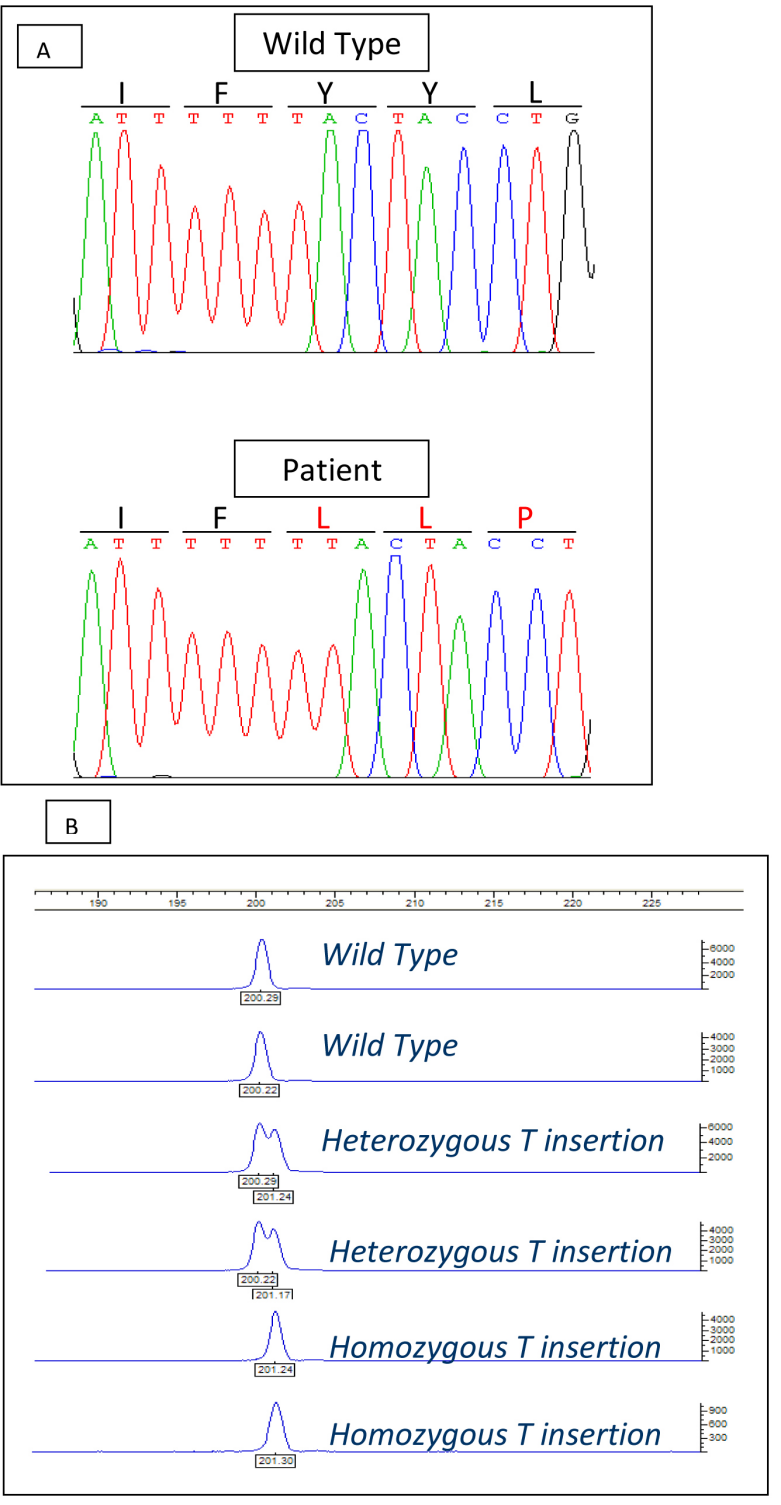
Molecular studies. **A**: Sequence chromatogram from a normal subject (top) and a CORD patient (bottom) with a homozygous insertion of T at position 1349 of the cDNA (c.1349insT) which results in a frame-shift starting at codon 452 and a putative stop codon 12 amino acids downstream in the translated protein (p.Y452fs12X). **B**: ABI 3100 assay for the detection of the *PROM1* mutation. The 1bp difference is evident in the homozygotes (reading peak at 201 bp) compared to controls (reading peak at 200 bp). Heterozygotes appear as two peaks.

The initial genome scan detected 11 microsatellite markers that shared homozygous allele readings in all 3 affected family members (D4S2935, D4S403, D6S158, D7S516, D7S484, D9S171, D9S287, D19S197, D11S925, D13S171, and D16S3091). Of these D4S2935, D4S403, and D7S516, D7S484 were consecutive, and therefore were thought to represent larger regions of homozygosity. When these regions were analyzed in the entire family with additional markers, only the chromosome 4p locus demonstrated full segregation. The 2.1 Mb critical interval was flanked by a telomeric obligate recombination in family member 4200103 for the marker UniSTS:1230568 (14.8 Mb) and a centromeric recombination in family member 4200105 for UniSTS:1230565 (16.9 Mb; Figure 1). The interval contains ten known genes including *PROM1* (15.57 Mb). Sequencing of the 26 coding exons of PROM1 in one of the patients disclosed a homozygous insertion in exon 12 (c.1349insT; Figure 4A), which results in a frame-shift starting at codon 452 and a putative stop codon 12 amino acids downstream in the translated protein (p.Y452fs12X).

The mutation was confirmed, then extended to other family members by visualization of the 1 bp difference on an automated ABI Prism 3100 Genetic Analyzer (Figure 4B).None of the other family members were homozygous for the mutation. Screening of 100 control chromosomes from Arab descent revealed only the wild-type allele.

## Discussion

We describe three siblings from a consanguineous Arab family who had central visual loss since childhood, night blindness, visual fields constriction, and high myopia. Electrophysiology studies and funduscopic examinations were consistent with CORD. A novel homozygous frameshift mutation was observed in PROM1. The mutation showed full segregation within the family and was not detected in a population of matched controls.

A striking feature of the disease in this family is the presence of axial myopia. High myopia which accompanies retinal dystrophies is characterized by an early age of onset, a high degree of refractive error, and is considered to have an underling hereditary etiology. It is plausible that the *PROM1* mutation may have contributed for both CORD and high myopia in this family. However absence of myopia in the other *PROM1*-related phenotypes and lack of *PROM1* expression in the sclera argues against *PROM1* involvement in the pathogenesis of the myopia. Another possibility is that the myopia and CORD are caused by mutations in two independent genes that are tightly linked. In such a case the neighboring gene fibroblast growth factor binding protein-1 (*FGFBP1*; OMIM 607737), located approximately 30 kb from *PROM1*, is a potential candidate gene for the observed myopia. A third possibility is that the myopia seen in our CORD patients may simply be induced by blurred vision. Vision deprivation has been reported to induce myopia in the chicken, mouse, and monkey [16,17]. Many previous studies have reported a high prevalence of myopia among patients with retinal dystrophy [18].

Interestingly, deleterious *PROM1* mutations were described by Maw et al. and Zhang et al. in 2000 and 2007 respectively, in autosomal recessive RP families [11,12]. The patients in these RP families differ from ours in several aspects. While visual night functions were initially spared in our patients, night blindness was the presenting symptom in the families described by Maw and Zhang, and they did not suffer photophoibia or myopia. Moreover, their fundus examination revealed typical RP findings of waxy-pale discs, obvious attenuation of blood vessels, and typical bone-spicule pigmentation in the mid-peripheral retina. Finally, in contrast to our family, their ERG recordings did not detect any rod responses. Despite these differences, the manifestations of all three families with deleterious *PROM1* mutations, demonstrate a gradually evolving rod and cone deterioration. All patients presented at childhood and experienced severe progression of the disease, an observation that emphasizes a key role for *PROM1* in the maintenance and function of rods and cones.

In contrast to the recessive inheritance mentioned, three families with a *PROM1* missense mutation (R373C) and autosomal dominant inheritance have also been described [13]. Two of these families had a Stargardt-like macular dystrophy (STGD4; OMIM 603786 [19]) and bull’s eye macular dystrophy (MCDR2; OMIM 608051 [20]), while a third family has only been mentioned briefly to have autosomal dominant cone-rod dystrophy without supplement phenotypic description.

Based on the data presented in our study, it is evident that mutations in *PROM1* are associated with a wide variety of symptoms ranging from mild autosomal dominant macular degenerations, via CORD, to severe autosomal recessive RP. How could this diversity be explained? The severe frameshift and null mutations observed in the recessive RP and CORD most likely abolish the function of one allele leading to lack of protein production from that allele. However a single functioning allele is sufficient to maintain normal retinal activity; only when both alleles are lacking do disease symptoms develop. In contrast, missense mutations result in a mutant protein that also interferes with the action of the normal protein, exerting a negative dominant effect and explaining the autosomal dominant inheritance. Indeed, immunohistochemistry studies in transgenic mice carrying the R373C mutation revealed that not only the mutant protein was mislocalized but it also caused the mislocalization of the wild-type protein. The mutant protein also impaired the function of two other proteins that are essential for photoreceptor integrity: Protocadherin 21 (PCDH21; OMIM 609502) [21] and the cytoskeletal actin filament [22]. The observed interactions between PROM1and other proteins involved in the photoreceptors disk formation and maintenance unravel the complexity of this process and may account, at least in part, for the phenotypic variation. Most likely additional background genes and environmental factors are also involved.

The mutation described in this report further expands the clinical spectrum of *PROM1* mutations.
